# Local full-thickness excision for sessile adenoma and cT1-2 rectal cancer: long-term oncological outcome

**DOI:** 10.1007/s00423-022-02593-7

**Published:** 2022-06-22

**Authors:** Maria A. Gascon, Vicente Aguilella, Tomas Martinez, Luigi Antinolfi, Javier Valencia, Jose M. Ramírez

**Affiliations:** 1 Department of Surgery, “Lozano Blesa” University Hospital, San Juan Bosco 15, 50009 Saragossa, Spain; 2 Department of Microbiology, Preventive Medicine and Public Health, University of Zaragoza, Domingo Miral s/n 50009-Saragossa, Spain; 3 Department of Radiotherapy, “Lozano Blesa” University Hospital, San Juan Bosco 15, 50009 Saragossa, Spain; 4 Aragon Health Research Institute, San Juan Bosco 13, 50009 Saragossa, Spain

**Keywords:** Transanal endoscopic microsurgery, Local surgery, Rectal cancer, Rectal adenoma, Oncological outcome, Rectal surgery

## Abstract

**Purpose:**

We analyzed all patients who underwent local transanal surgery at our institution to determine oncological outcomes and perioperative risk.

**Methods:**

In 1997, we developed a prospective protocol for rectal tumors: transanal local full-thickness excision was considered curative in patients with benign adenoma and early cancers. In this analysis, 404 patients were included. To analyze survival, only those patients exposed to the risk of dying for at least 5 years were considered for the study.

**Results:**

The final pathological analysis revealed that 262 (64.8%) patients had benign lesions, whereas 142 had malignant lesions. Postoperative complications were recorded in 12.6%. At the median time of 21 months, 14% of the adenomas and 12% of cancers had recurred, half of which were surgically resected. The overall 5-year survival rate was 94%.

**Conclusion:**

With similar outcomes and significantly lower morbidity, we found local surgery to be an adequate alternative to radical surgery in selected cases of early rectal cancer.

## Introduction

The excision of rectal adenoma is accepted worldwide as rectal adenoma is a premalignant condition. Moreover, unexpected rectal cancer may be encountered incidentally in the resected specimen despite preprocedural diagnostics [1]. As such, the removal of rectal adenomas relieves symptoms and lowers the incidence of carcinomas. When possible, these lesions are best treated with snare polypectomy; however, they will need surgery in some cases.

Local rectal surgery was considered only for benign tumors with interest in local excision for rectal cancers beginning only in earnest after Morson et al. [2] published their experience of the conventional transanal approach in 1977, which demonstrated a low rate of local recurrence in early distal tumors when excised intact with negative margins.

In 1983, the transanal endoscopic microsurgery (TEM) system was introduced as a technique to ease local rectal surgery [3]. The first results published by TEM pioneers have shown it to be technically superior to the conventional peranal approach, with significantly lowers recurrence rates and an ability to access the entire rectum [4, 5]. The TEM technique gained widespread support during the latter years of the last century and renewed the interest in local rectal surgery among surgeons. Due to this renewed interest [6], an innovation race began, and in a few years, different systems appeared, each one claiming some advantage over the rest [7, 8].

In this sense, as its use becomes popular, considering Morson’s criteria for local excision and recalling the value of the experience of the surgical team are essential to ensure an optimal outcome. We conducted a prospective study on the TEM technique for sessile rectal adenomas and early rectal cancer; the primary aims of this study were perioperative morbidity and long-term oncological survival. The protocols and first oncological results (1997–2006) have been reported previously [9, 10]. Such protocols and the prospective recording data have remained the same.

In this study, we performed a final analysis of all patients with rectal lesions who underwent local surgery with a curative intent according to the strategy established in 1997 at our institution, a high-volume TEM academic center. The aim was to outline the perioperative risk and long-term oncological outcome of this strategy and to guide surgeons and patients in the shared decision-making process.

## Materials and methods

This study included all patients entered into our prospective database because of a rectal lesion who underwent local surgery. The inclusion criteria according to our protocol were as follows: adult patients with benign adenoma unsuitable for endoscopic resection and stage I low-risk (LR) rectal cancer. In cases of high-risk (HR) pT1 or pT2, adjuvant radiotherapy would be offered as an alternative to radical surgery. The protocol was approved in due time by our Institutional Ethics Committee.

### Preoperative evaluation

After detailed history taking, physical examination was complemented by digital rectal assessment and rigid rectoscopic examination to estimate the location and distance from the anal verge. We only selected TEM with a curative intent for tumors located in the extraperitoneal region of the rectum that could be removed with sufficient free margins.

Endorectal ultrasonography (ERUS) was performed to stage the neoplasms. Moreover, preoperative evaluation included full colonoscopy to rule out synchronous lesions and obtain samples for histopathological evaluation.

For patients with biopsy-proven adenocarcinoma, the study was complemented with carcinoembryonic antigen (CEA) determination, chest X-ray, and abdominopelvic computed tomography (CT). Moreover, pelvic magnetic resonance imaging (MRI) was used from 2003 onward.

After this diagnostic workup, patients with benign lesions were scheduled for local surgery. Those with cancer were presented to a dedicated multidisciplinary team. Before the procedure, patients were counseled in detail about the benefits and risks involved, and written informed consent was obtained.

### Surgery, pathology, and treatment options

All patients underwent full bowel preparation and received short-term antibiotic prophylaxis. General anesthesia was used in all cases. All procedures were performed by two senior surgeons (VAD and JMRR)—both were former trainees at Professor Büess’ training center in Tübingen (Germany). TEM was performed using equipment from Richard Wolf (Knittlingen, Germany). A more detailed description of the instruments and technique has been reported previously [11]. The traditional transanal approach, using conventional instruments (Hill Ferguson or Parks retractors), and direct visualization were indicated for the lowest lesions, which were tumors located between the dentate line and the first rectal valve. The institutional strategy for all these lesions is full-thickness excisions with grossly negative 1-cm peripheral margins. During the early years of TEM in our institution, suturing the defect was an option; however, after the results of our randomized study, this step was no longer routinely performed, and the decision was based upon the surgeon’s preference [12].

Resected specimens were pinned on a cork plate, measured, and sent to the pathology department for examination. Surgical excision was considered complete after confirmation of full-thickness excision and free circumferential margins (≥ 1 mm) on microscopic evaluation.

**Fig. 1:**
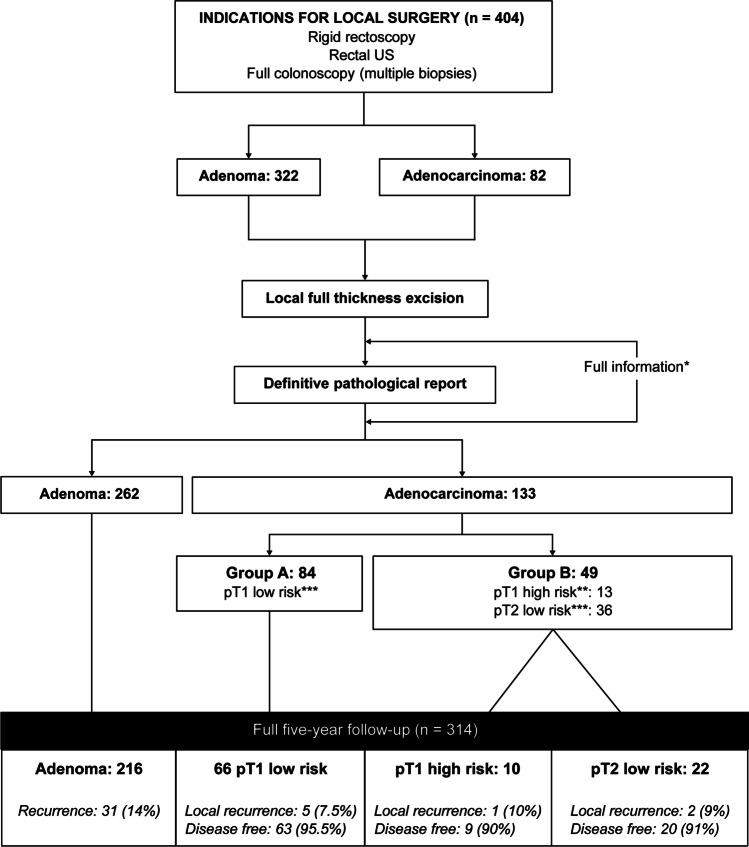
Distribution and outcomes of the patients according to our protocol. *Full information is given to the patient before and after surgery for shared decision-making. **In cancers, high risk was defined if any of the following characteristics were mentioned in the pathology report: poor differentiation, lymphatic or venous invasion, or a clear resection margin of less than 1 mm. ***Low risk was defined when the cancers did not have any of the high risk characteristics

Rectal cancer was defined as HR if any of the following characteristics were mentioned in the pathology report: poor differentiation, lymphatic or venous invasion, and a clear resection margin of < 1 mm. According to the final pathological report (Fig. 1), the patients were grouped as follows:

Group A: Benign or pT1 LR lesions. TEM alone was considered curative, and no further treatment was required.

Group B: pT1 HR or pT2 LR. In these cases, after being fully informed about the merits and disadvantages of both treatment modalities, the patients were offered either salvage surgery or adjuvant radiotherapy (5040 cGy in 28 fractions).

### Follow-up

The patients were followed up at the outpatient clinic and underwent physical examination, rigid proctosigmoidoscopy, and ERUS for the first time at 4 weeks, every 3 months thereafter for 2 years, every 6 months up to the 5th postoperative year, and yearly thereafter. For patients with malignant neoplasms, the follow-up was complemented by the evaluation of serum CEA levels and regular thoracoabdominal CT. Moreover, the follow-up regimen included full colonoscopy 1 and 3 years after local surgery.

### Statistical analysis

The statistical analysis was descriptive and is indicated using means and standard deviations. Differences between groups were determined using the Mann–Whitney *U*-test and chi-square test. Overall and cancer-specific survival curves were estimated using the Kaplan–Meier method. The log-rank test was used to examine differences in outcomes. Multivariate analysis was performed using the Cox proportional hazards model. Differences with *p-*values < 0.05 were considered statistically significant. All analyses were performed using R (R Foundation for Statistical Computing, Vienna, Austria).

## Results

In the study period between January 1997 and December 2017, 404 patients were enrolled who underwent local surgery with curative intent (Fig. 1). Of these, 237 (58.7%) were men, with a mean age of 68 years (range, 20–92 years). With regard to preoperative clinical staging, 322 (79.7%) patients had adenoma, and 82 (21.3%) had stage I rectal cancer. The median upper distance from the anal verge was 9.7 cm, and the mean size of the lesions was 3.7 cm (Table 1).

**Table 1:** Pathological and operative characteristics of the patients

*Variable*	*All* *(n* = *404)*	*Adenoma* *(n* = *262)*	*Carcinoma* *(n* = *142)*	*P-value*
*Mean age, years (SD)*	*68 (11.9)*	*67.8 (12.1)*	*68.5*	*0.99*
*Male sex (%)*	*237 (58.7)*	*153 (58.4)*	*84 (59.1)*	*0.97*
*Mean lesion size, cm (SD)*	*3.68 (1.60)*	*3.73 (1.63)*	*3.58 (1.55)*	*0.48*
*Mean upper distance from anal verge, cm (SD)*	*9.7 (3.9)*	*9.6 (4.0)*	*9.8 (3.5)*	*0.44*
*Defect closure (%)*	*145 (35.9)*	*86 (32.8)*	*59 (41.6)*	*0.10*
*Tumor fragmentation (%)*	*10 (2.5)*	*6 (2.3)*	*4 (2.8)*	*0.10*
*Mean duration of surgery, min (SD)*	*78 (45.7)*	*76.7 (46.6)*	*80.3 (43.9)*	*0.16*
*Postoperative complications (%)	*51 (12.6)*	*37 (14.1)*	*14 (9.9)*	*0.28*
*Postoperative bleeding (%)*	*32 (7.9)*	*22 (8.4)*	*10 (7.0)*	*0.76*
*Positive margin (%)*	*21 (5.2)*	*18 (6.9)*	3 (2.1)**	*0.06*
*Mean length of stay, days (SD)*	*4.4 (4.05)*	*4.5 (4.6)*	*4.2 (2.8)*	*0.54*

^*^Some patients had more than one complication

^**^According to our protocol, patients with cancer with positive margins were considered high risk and received adjuvant radiotherapy

### Surgical procedure and histopathological examination

Of the 404 patients enrolled in this study, TEM was performed in 367 (91%), with the traditional transanal approach being performed in the remaining 36 (9%) cases; a deep full-thickness excision was performed in all cases.

A peritoneal defect was noted intraoperatively in six cases; the suturing of the defect in one of the cases required abdominal laparoscopic assistance. The tumor was extracted fractioned in 10 (2.5%) cases due to technical difficulties. At the end of the operation, the defect was left open in 259 (64%) patients.

The median operation time was 78 min (range: 50–90 min). The final surgical pathological analysis (Table 1) revealed that 262 (64.8%) patients had benign lesions and 142 (35.2%) had malignant lesions (97, pT1; 37, pT2; and 8, pT3). Table 2 shows the relationship between preoperative ERUS and the final pathological report. The overall accuracy of preoperative ERUS was 77% (64% for malignant T1 lesions). Of 45 patients thought to have T2 lesions, 20 (44%) were misdiagnosed (12 overstaged and 8 understaged).

**Table 2:** Ultrasound vs. definitive histological report

	All	Benign	pT1	pT2	pT3
	*N* = *404*	*N* = *262*	*N* = *97*	*N* = *37*	*N* = *8*
uT0	302	254	48	–	–
uT1	57	4	37	16	–
uT2	45	4	12	21	8

As reported previously, preoperative biopsy and ERUS could not appropriately differentiate between benign lesions and early cancer or between the levels of wall invasion or grading, both of which are of particular interest in the decision-making process. In any case, according to our proposal strategy, only 9 (2%) of the 404 patients were incorrectly included for local surgery (1 with pT2 HR and 8 with pT3).

According to our policy, all these subjects underwent immediate radical surgery, as did three patients from group B after the final shared decision-making process.

Regarding margins, in 21 patients (5.2%), the dissection was considered microscopically incomplete or doubtful because the normal circumferential margin was smaller than 1 mm, whereas there were no patients whose deep margins were compromised.

No statistical differences were observed between adenomas and carcinomas in terms of histological findings according to age, gender, tumor size, distance to the anal verge, or location (Table 1).

### Postoperative complications

Postoperative complications were recorded in 51 (12.6%) of the 404 patients, some of whom had more than one complication. Bleeding was the most common early complication (Table 1), occurring in 32 (8%) cases. Nine patients with bleeding needed repeat TEM for hemostasis (two of them also required transfusion).

Postoperatively, one patient (0.25%), a 76-year-old man, underwent the Hartmann procedure on the fourth postoperative day because of intraabdominal sepsis owing to an undetected leakage. Despite this, the patient died 20 days after the reoperation.

### Recurrence and survival status

As the main outcomes were long-term recurrence and survival after local surgery, data in this study were based on the full 5-year follow-up. After the first postoperative year, 41 (10%) of the 404 patients were lost to follow-up: one postoperative death and 40 patients (26 with adenomas and 14 with cancer) for reasons unrelated to the surgery. As we analyzed the oncological outcomes of local surgery, we excluded the 12 patients who underwent immediate radical surgery, understanding local surgery as biopsy that appropriately and accurately staged the patient.

Of the remaining 351 patients, 314 (216 adenomas and 98 malignant tumors) met the minimal established follow-up period.

#### Adenoma recurrence

A total of 31 (14%) of 216 patients with benign lesions demonstrated recurrence at a median duration of 21 months (interquartile range [IQR], 15–28 months). Of the 31 patients, 22 were managed with the new TEM technique, and nine were treated with snare polypectomy. Recurrence for the second time was observed in eight patients (median time, 15 months; IQR, 14–24 months), and recurrence for the third time was observed in one case (12 months after the second). Recurrence was not statistically related to gender, age, margin affected, fragmentation, or defect closure; it seems to be related to the dysplasia grade (Table 3). No recurrence was observed in patients who underwent surgery by conventional transanal approach.

**Table 3:** Risk of recurrence of adenomas and carcinomas

*Variable*	Adenomas*	Carcinomas*	OR (univariate)**
*Gender-Male*	0.69 (0.34–1.39, *p* = 0.301)	0.77 (0.31–1.94, *p* = 0.579)	1.05 (0.65–1.71, *p* = 0.840)
*Age*	0.98 (0.95–1.00, *p* = 0.080)	0.99 (0.95–1.04, *p* = 0.791)	1.00 (0.98–1.02, *p* = 0.774)
*Tumor size*	1.19 (1.00–1.41, *p* = 0.050)	0.86 (0.61–1.23, *p* = 0.415)	0.98 (0.84–1.14, *p* = 0.818)
*Fragmentation*	1.58 (0.22–11.61, *p* = 0.651)	0.00 (0.00–Inf, *p* = 0.998)	2.26 (0.52–9.72, *p* = 0.257)
*Non clear margins*	2.27 (0.79–6.49, *p* = 0.126)	3.65 (0.84–15.93, *p* = 0.085)	0.46 (0.10–1.44, *p* = 0.225)
*Closure of the defect*	0.58 (0.25–1.35, *p* = 0.205)	0.71 (0.27–1.89, *p* = 0.489)	1.38 (0.85–2.25, *p* = 0.194)
*Postop. complication*	1.23 (0.37–4.05, *p* = 0.732)	0.00 (0.00–Inf, *p* = 0.998)	0.63 (0.20–1.64, *p* = 0.372)
*Low-mod. dysplasia*	0.35 (0.13–0.92, *p* = 0.034)		–
*High dysplasia*	0.80 (0.34–1.90, *p* = 0.620)		–
*pT2*		0.79 (0.23–2.71, *p* = 0.703)	

^*^Cox regression

^**^Odds ratio univariate logistic regression

#### Carcinoma recurrence and survival

Of the 98 patients who underwent local excision of malignant lesions with a curative intent and had a minimum follow-up of 5 years, 66 (67.4%) were classified into group A and 32 (32.6%) into group B (Fig. 1). During the follow-up, seven patients with a pathological report of pT1 presented a rectal benign lesion (severe dysplasia), which was excised using the new TEM technique.

**Fig. 2:**
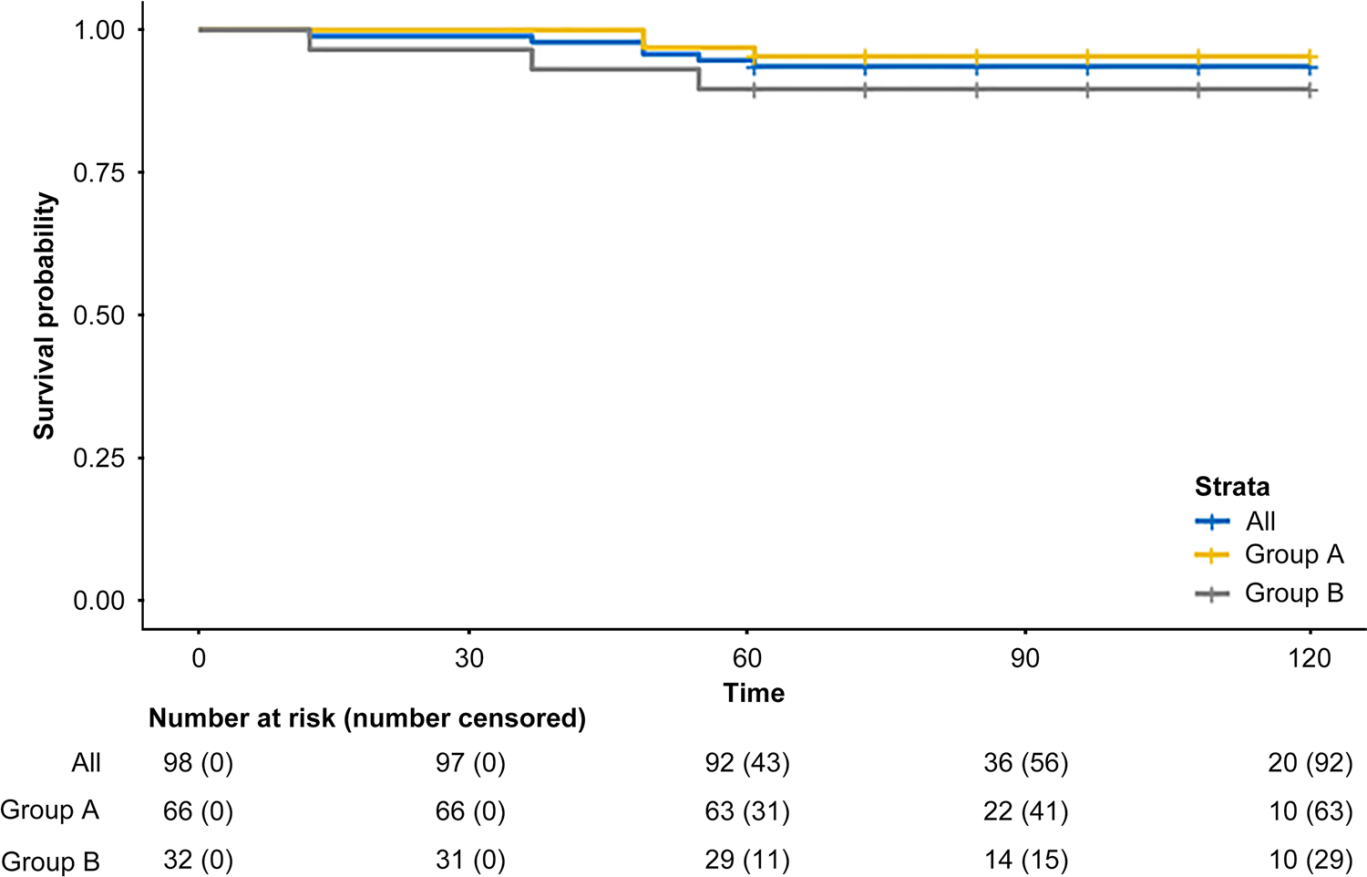
Disease-free survival curve (*n* = 98) stratified by groups

Table 4 shows protocol failures. Eight of the 98 patients with cancer (Table 4; Fig. 1) presented local cancer recurrence (five from group A and three from group B), at the median duration of 16.5 months (IQR, 14–31 months). In all cases, radical resection was possible; however, three patients died of cancer. Without local recurrence, one patient presented with periaortic lymph node involvement (died from the disease) and three had liver metastases (only one of them was disease-free after partial hepatectomy). The overall cancer-related mortality was 6.1%: three of the 66 patients from group A (4.5%) and three of the 32 from group B (9.4%) died. The 5-year disease-free survival curve from both groups is displayed in Fig. 2.

**Table 4:** Protocol failures (*n* = 98)

	Age	Gender	Site of recurrence	Months to recurrence	Procedure (PR)	Status
*pT1 low risk*	73	Male	Local	15	AAP (pT1N0)	Free from disease
*pT1 low risk*	46	Male	Local	39	LAR (pT2N0)	Free from disease
*pT1 low risk*	60	Female	Local	30	AAP (pT2N1)	Free from disease
*pT1 low risk*	69	Female	Local	19	AAP (pT2N0)	Free from disease
*pT1 low risk*	49	Female	Periaortic node	24	Lymphadenectomy	Death
*pT1 low risk*	70	Male	Liver	36	Hepatectomy	Free from disease
*pT1 low risk*	83	Male	Liver	42	Adjuvant therapy	Death
*pT1 low risk*	57	Female	Local	36	AAP (pT2N1)	Death
pT1 high risk*	69	Male	Local	12	AAP (pT3N1)	Death
pT2*	61	Female	Local	15	LAR (pT2N1)	Free from disease
pT2*	60	Male	Local	12	AAP (pT3N1)	Death
pT2*	68	Female	Liver	24	Hepatectomy	Death

*AAP* abdominoperineal resection, *LAR* low anterior resection, *PR* pathological report

^*^Patients with adjuvant radiotherapy

## Discussion

Endoscopic techniques have improved over time, and a significant proportion of rectal adenomas are now being resected using endoscopic mucosal resection. TEM was originally developed to be complementary to endoscopic mucosal resection and is particularly useful for treating large villous adenomas, for which it remains indicated. Given the low associated morbidity and lack of mortality compared with radical surgery, a proposal has been made to extend this indication to cases of rectal cancer with low probability of lymph node involvement and local recurrence. Although early rectal cancer is relatively uncommon in the West, the generalization of screening programs has indicated that nearly 50% of tumors detected are stage I (T1–2 N0) tumors [13].

Total mesorectal excision (TME) provides the best long-term prognosis for rectal cancer, with low rates of local recurrence and excellent long-term survival. However, this accomplishment is not without an important degree of postoperative morbidity and compromised quality of life [14]. Moreover, permanent stoma rates of up to 37% have been reported [15]. Indeed, balancing the level of surgical morbidity and mortality for all stages of resectable rectal cancer against a satisfactory oncological outcome is a challenge, although, based on current evidence, it appears that TME could be considered overtreatment in some early rectal cases [10, 16]. In this regard, current clinical guidelines agree that, for LR pT1 rectal cancer, local excision is deemed sufficient, whereas for HR pT1 or pT2, adjuvant (chemo) radiotherapy may be an alternative to TME within a clinical trial setting or for patients unfit for surgery [17]. As such, the key point is to perform an accurate preoperative staging, a difficult task that still relies mainly on two imaging tests, namely, ERUS and pelvic MRI scan. ERUS remains the gold standard for evaluating the depth of wall penetration [18], and many clinicians believe that ERUS contributes widely to the management of patients with early disease. However, recent findings have shown that ERUS is not a reliable technique for distinguishing between adenoma and T1 and has an important rate of inaccuracy for determining T1 and T2 [19, 20]. Unfortunately, MRI does not provide any advantages in this regard, and overstaging tends to occur: MRI results are impressive in advanced disease but less accurate in defining early invasion [20, 21]. All this is of paramount importance as overstaging rectal tumors by ERUS or MRI may result in a decision to use radical instead of local surgery, thus denying patients with early disease a potentially curative and more minor operation.

In our series, 322 of 404 tumors had biopsy of adenoma. From a theoretical viewpoint, all these would be suitable for a mucosectomy or partial-thickness excision. Sixty (19%) of them were cancers, and the full-thickness excision technique helped avoid a second local surgery or, even worse, TME. In this context, some authors have highlighted that patients with rectal sessile tumors without invasive carcinoma on biopsy and without malignant characteristics in the judgment of an experienced colorectal surgeon might not benefit from preoperative imaging before undergoing transanal local surgery [22].

Taking a closer look at the 82 biopsy-proven cancers is interesting as 16 uT1 were already pT2 (Table 2) and were, according to the guidelines, undertreated by local surgery. In these cases, additional treatment (completion TME surgery or adjuvant chemoradiotherapy and close surveillance) should be considered in the shared decision-making process. A recent study indicated that there is no oncological loss from performing local excision before completion of surgery; however, there may be an increased risk for a permanent stoma [23].

In contrast, 11 uT2 cases were already pT1 LR; therefore, these cases would have had unnecessary radical surgery (TME or abdominoperineal resection with permanent colostomy) if we had followed the guidelines.

At the time, we proposed our protocol—postoperative radiotherapy—was the standard for managing rectal cancer and was recommended after local excision of T1 with poor histological features and T2 [24, 25]. Indeed, we used it as a routine procedure in some cases with only minor problems, all of which were treated conservatively. Nowadays, there is an increasing tendency to use neoadjuvant chemotherapy or radiation therapy, followed by TEM, an issue due to the lack of accuracy in preoperative staging.

TEM is a surgical technique linked to few complications, with postoperative bleeding being the most common (0.5–4.1% of cases) [26, 27]. Additional risks communicated to patients include anal incontinence, rectal stenosis, and rectovaginal fistula. Overall, our complication rate was 12%, which is similar to those reported in previous studies on TEM in the literature (5–21%) [28, 29]. However, this technique is not free from mortality, and one patient in our series died (0.2%); this rate is similar to that reported by other authors [30]. Thus, morbidity after TEM is significantly lower than that reported after radical surgery; however, recurrence is its Achilles heel.

Pigot et al. [31] demonstrated that, in large rectal tumors, the risk of recurrence of benign polyps was 10%, whereas if a malignancy was identified, the risk increased to 20%. The risk factors for adenoma recurrence include size, previous piecemeal excision, and positive resection margin [32]. Moreover, unsutured management of the defect has also been identified as a risk factor for recurrence [33]. In our experience, after a minimum surveillance period of 5 years, the recurrence rate of adenomas was 14%. We could not statistically demonstrate any independent factor for recurrence; however, the dysplasia grade seemed to be an independent risk factor. In line with previous reports, leaving a full-thickness rectal defect open seems to be safe and does not show any difference in complication or recurrence rates [12, 34].

After the local treatment of rectal cancer, ensuring the early detection of recurrent disease is of paramount importance. Based on our data, the most intense surveillance should be performed within the first 3 years. As recommended by some authors, locally excised rectal cancers should have specific surveillance guidelines, including periodic pelvic MRI and proctoscopy [35].

Accurately determining the 5-year survival is difficult after radical surgery for stage I rectal cancer as this should consider both cancer-related and postoperative mortality. Moreover, although perioperative patient management has improved recently, the rectal cancer population has aged. In any case, the accepted range is 90–95% [36, 37]. Recently published studies have presented similar rates of survival after local excision of pT1 and pT2 plus adjuvant therapy [38, 39]. In our fully controlled patients, we encountered six cancer-related deaths, thus providing a survival rate of 94% (95.5% for pT1 and 90% for pT2 plus radiotherapy).

Minimally invasive instrumentation is continually evolving, and local rectal excision can currently be performed using the transanal approach, TEM equipment, transanal minimally invasive surgery [40], or even robotic surgery [41]; whatever the technique, surgeons must be part of a multidisciplinary specialized team prepared to discuss any particular case and translate this debate to patients to incorporate their opinions into the decision-making process. Patients should be engaged in a partnered dialog in which the actual risks and benefits of treatment options are presented.

This study has several limitations. First, this was a retrospective analysis of prospective data from a consecutive series of patients who underwent local surgery over a prolonged period. Results could be compromised due to inherent technical evolution.

## Conclusion

With similar outcomes and significantly lower morbidity, we found local surgery (alone or adding radiotherapy) to be an adequate alternative to radical surgery in selected cases of early rectal cancer (pT1–pT2). Patients should be involved in the decision-making process and should know the actual risks and benefits of the treatment options.
